# Impulsivity: A Predisposition Toward Risky Behaviors

**DOI:** 10.5812/ijhrba.20428

**Published:** 2014-06-01

**Authors:** Nour-Mohammad Bakhshani

**Affiliations:** 1 Department of Clinical Psychology, Children and Adolescent Health Research Center, Zahedan University of Medical and Sciences, Zahedan, IR Iran

**Keywords:** Impulsive Behavior, Dangerous Behavior, Risk-Taking

The construct of impulsivity is significantly important in research and clinical fields concerning risky behaviors and some mental disorders. Although, according to DSM and ICD, impulsivity is recognized as a diagnostic criterion for several disorders (1), but the way it influences mental disorders and development of risky behaviors is not yet clear. Disagreement over the definition of impulsiveness, its main components, and measurement techniques could be considered as major causes that make forming a comprehensive theory regarding impulsivity development and its role in psychopathology impossible.

Impulsivity has been investigated from various perspectives and several definitions have been proposed. From a charachterological perspective, Eysenck holds that impulsivity is characterized by unplanned risky behaviors, and making up one’s mind quickly (2). Dickman proposed that dysfunctional impulsivity is characterized by taking action with less thought in comparison to most individuals with the same level of skill and knowledge. Later, Dickman pointed out another component called inhibition component which manifests as inadequate attention, which itself is a cause of impulsivity (3, 4). Barratt distinguished three dimensions of impulse:

- motor (action without thinking),
- cognitive (quick cognitive decision-making), and
- non-planning (decrease in orientation towards future) factors (5).

Nigg et al*.* defined impulsivity as a rash response in situations where considerate response is more appropriate (6). According to Patton et al*.* there are three factors contributing to impulsivity:

- acting on the spur of moment (motor activation),
- not focusing on the task at hand (inattentiveness) , and
- not planning and thinking carefully (non-planning) (7).

From a biological and neuropsychological perspective, impulsivity is characterized by failure in inhibiting a potentially risky impulse for the individual or the others around (8). From a cognitive viewpoint, impulsivity is the inability to inhibit behavioral impulses and thoughts. It considers impulse control as an important component of executive functions. It plays an important role in one's social and personal functioning (9). Considering social aspect of impulsivity, it referred as a learned behavior that is formed inside the family. In family, children learn to react immediately in order to achieve what they desire (10). Therefore, impulsive individuals lack the ability to evaluate the consequences of their actions, either for themselves or for the others.

Moeller et al*.* look upon impulsivity from a bio-psycho-social perspective in order to incorporate various cognitive-social and its characterological aspects. They hold that a comprehensive definition of impulsivity should include all these aspects (1):

1. Decreased sensitivity to negative consequences of behavior.
2. Immediate and unplanned reaction to stimuli before processing the information thoroughly.
3. No regard for long-term consequences of a behavior.

Moeller et al*.* (1) believed that impulsivity includes readiness to take immediate and unplanned action as a response to internal and external stimuli, with no regard for their negative consequences for themselves or the others. This definition has several features, which are helpful in research and treatment. Firstly, impulsivity is considered as a potential that itself could be considered as a part of behavioral pattern. Secondly, impulsivity includes an immediate and unplanned action that takes place while there has not been enough chance for evaluating the consequences. According to this feature, impulsivity is distinguished from impaired judgment or compulsive behavior in which planning has already taken place before the action. Thirdly, impulsivity indicates taking action with no regard for its consequence.

Among cognitive theories, Kagan's theory has had a significant influence on learning and processing theories. Kagan considered behavioral inhibition as a temperament in children, which influences behavioral, cognitive and psychological responses to novelty (11). From a behavioral perspective, impulsivity includes a wide variety of actions that , are immature, dangerous, inappropriate to the situation and done without consideration, which usually bring about negative consequences (12). Impulsive individuals can not delay their satisfaction and control themselves (13). When faced with various consequences, they prefer less worthy immediate rewards to delayed more worthy ones (14). Ho et al*.* include punishment in their definition of impulsivity, that is preferring smaller short term gains to delayed bigger ones, or preferring bigger delayed punishments to immediate small ones (15).

However, motor (behavioral) impulsivity is distinguished from cognitive impulsivity (selection). Motor Impulsivity is equal to response inhibition and it is studied on animals (16-18). This type of impulsivity is related to dorsolateral prefrontal lobe (19). Moreover, the role of 5-hydroxytryptamine in animal's aggression and addiction is important (20). On the other hand, cognitive impulsivity is characterized by inability to compare immediate consequences and the future of events with each other, and consequently, the inability to delay satisfaction. This type of impulsivity is evaluated in decision-making tasks (21). Ventromedial prefrontal lobe is recognized as the main part involved in this kind of impulsivity (22).

Brunner and Hen (20) distinguish impulsive action (behavior) from impulsivity (basic psychological processes). In impulsive action, when an individual faces two reward choices, one of which is small but immediate, and the other one is big but delayed, he/she chooses the former (impulsive action). It is because they cannot delay their satisfaction (impulsivity). However, if an individual chooses the first choice due to his/her inability to evaluate and compare the rewards against one another, though his behavior is still impulsive, the psychological process that controls his behavior is not his/her inability to delay satisfaction, but it is his inability to distinguish between the two choices.

In psychopathology, impulsivity is defined in three different ways:

- Fast reaction without thinking and conscious judgment,
- acting without enough thinking, and
- a tendency to act with less thinking compared to the others who have similar levels of knowledge and ability (23).

In general, impulsivity is the symptom of some disorders, such as hyperactivity disorder (24), depression and anxiety disorder (25), and personality disorders, especially cluster B disorders (antisocial personality disorder and borderline personality disorder). According to DSM-V, impulsivity is defined in terms of an aspect of disinhibition, and considered as an immediate reaction to stimuli, unplanned reaction on the spur of the moment or with no regard for its consequences, problem in programming or adhering to programs, sense of urgency and self-harming behavior in the time of emotional turmoil (26). This clinical conceptualization only includes negative and pathological aspects of impulsivity, and does not distinguish between impulsivity and aggression. In order to account for the positive aspects of impulsivity, Dickman (3) distinguished between functional impulsivity and dysfunctional impulsivity. Dysfunctional impulsivity is present in several psychiatric disorders that bring about risky behaviors, including bipolar disorder, eating disorders, personality disorders, drug addiction, and ADHD.

The factor analysis of Barratt impulsivity scale indicates three factors: increased motor activity, decreased attention, and decreased planning. Decreased attention and planning are the main factors of impulsivity. The psychiatric disorders that come along impulsivity are probably related to different patterns of these basic mechanisms. For instance, frontal lobe lesions that impair attention and planning lead into the emergence of personality disorder symptoms, and a high level of motor activity is observed in mania. Moreover, the level of impulsivity is high in antisocial individuals, but its level is different. Linnoila et al. found out that compared with individuals with preplanned violence, impulsively violent individuals have a lower level of serotonin metabolite in their cerebrospinal fluid (CSF) (27). In addition, Coccaro et al*.* reported a significant correlation between prolactin response to fenfluramine, which causes the release of serotonin, and impulsive aggression in individuals with personality disorders. Frontal lobe lesion is mostly considered as a cause of impulsivity (28). Bechara et al*.* reported that patients with prefrontal lobe lesion are impaired in distinguishing between the choices with a positive consequence and the ones with a negative consequence (19).

Several studies have shown a correlation between committing suicide and impulsivity in the patients with borderline personality disorder. Soloff et al*.* reported a higher level of impulsive aggression in borderline personality disorder (29, 30). They also hold that it also predicts suicide patterns. Individuals with personality disorders with a history of attempted suicide, compared to individuals with no history of attempted suicide, showed a higher level of impulsive actions. Studies that investigated impulsivity in individuals with drug addiction, confirm the correlation between impulsivity and drug abuse. Individuals who abuse multiple drugs show a higher level of impulsivity, compared with individuals who abuse only one kind of drug (31, 32). Individuals with a history of consuming drugs prefer immediate rewards when faced with multiple choices of rewards, even if the reward is slight (33). Rarely can we see a manic episode without impulsive actions. Furthermore, in depressive episodes, the level of impulsivity is high, especially when there is a tendency to committing suicide.

The ability to choose appropriate actions significantly correlates with preplanning ability and response inhibition. Several studies showed that the prefrontal lobe is involved in some aspects of impulsivity. It is also confirmed that the processes involved in decision-making and mechanisms concerned with impulsivity, take place in specific parts of the prefrontal lobe (19).

The definition of impulsivity should consider various factors to facilitate a better understanding. In other words, several psychological processes may lead to impulsive behaviors, such as inability to store multiple choices in memory in order to evaluate them (working memory), or inability to predict actions. However, the problem of giving a single definition for impulsivity still exists. Investigating, also, the correlation between impulsivity, decision-making, and risky behaviors needs more research. Developments in this regard will contribute to enhance services providers to individuals with psychiatric disorder or with brain lesions, which is significantly important in decreasing risky behaviors.

Despite the many studies carried out regarding impulsivity, and its related bio-psycho-social biological variables in recent years, little work has been done to integrate the extensive data in this field. This is because there is no exact and unique definition of impulsivity, and there is no agreement over its major components. Due to these situations, there is no comprehensive theory regarding the development of impulsivity, and its interaction with internal and external stimulants. Therefore, in order to find out how impulsivity causes risky behaviors in different situations, it is needed to measure each impulsivity component and their role in the emergence of risky behaviors more precise. Secondly, more research should be carried out regarding designing and implementing programs to prevent and treat impulsivity.
